# Paternal Depression: An Examination of Its Links with Father, Child and Family Functioning in the Postnatal Period

**DOI:** 10.1002/da.20814

**Published:** 2011-04-19

**Authors:** Paul G Ramchandani, Lamprini Psychogiou, Haido Vlachos, Jane Iles, Vaheshta Sethna, Elena Netsi, Annemarie Lodder

**Affiliations:** Department of Psychiatry, University of OxfordOxford, United Kingdom

**Keywords:** depressive disorder, perinatal psychiatry, childhood experience

## Abstract

*Background*: Maternal depression is common and is known to affect both maternal and child health. One of the mechanisms by which maternal depression exerts its effects on child health is through an increased rate of parental disharmony. Fathers also experience depression, but the impact of this on family functioning has been less studied. The aim of this study was to investigate the association between paternal depressive disorder and family and child functioning, in the first 3 months of a child's life. *Methods*: A controlled study comparing individual and familial outcomes in fathers with (n=54) and without diagnosed depressive disorder (n=99). Parental couple functioning and child temperament were assessed by both paternal and maternal report. *Results*: Depression in fathers is associated with an increased risk of disharmony in partner relationships, reported by both fathers and their partners, controlling for maternal depression. Few differences in infant's reported temperament were found in the early postnatal period. *Conclusions*: These findings emphasize the importance of considering the potential for men, as well as women, to experience depression in the postnatal period. Paternal symptoms hold the potential to impact upon fathers, their partners, and their children. Depression and Anxiety, 2011. © 2011 Wiley-Liss, Inc.

## INTRODUCTION

Depression affecting parents is a common and important condition. An estimated 13% of women experience depression following childbirth,[1] and this can have an enduring effect on both the mother's health and their child's development.[2,3] There is now a substantial body of research showing consistent associations between maternal postnatal depression and an increased risk of cognitive, emotional, and behavioral problems in offspring.

More recent work has demonstrated that depression also affects 5–10% of fathers[4] and that it is associated with an increased risk of behavioral and cognitive difficulties in children.[5]–[7] This effect is found to be independent of the impact of maternal depression and may be particularly potent when the depression occurs very early in the child's life, in what may be a sensitive period of development. To date there has been only very limited research examining the impact of paternal depression on family life and child development in the postnatal period. This omission is important given the potential importance of the early months of a child's life for its subsequent development, and the importance of the impact of the couple relationship and maternal mental health as possible pathways by which risk is transmitted.[8] Studies of families with older children have demonstrated relatively consistent associations between parental depression and the couple relationship, with interactional processes occurring, whereby couple conflict is predicted by, but also predicts, parental depression.[9] A number of theoretical models have been proposed for these processes, but empirical research, particularly involving paternal depression, as well as maternal, is relatively lacking. Depressed men are more likely to express, and elicit, negative emotions in interactions with their partners, and also more likely to disengage from couple interactions at times of stress.[10,11] Men are also noted to report some different symptoms when suffering with depression, with higher levels of irritability and anger expressed.[12] These factors may lead to differences in the impact of depression in fathers on their children's development, compared to maternal depression, particularly in the early stages of child development.

In this study we sought to investigate the associations between paternal depression and parental couple functioning and child temperament in the first months of a child's life.

## MATERIALS AND METHODS

### SAMPLE AND PROCEDURE

The participants were recruited from the John Radcliffe General Hospital in Oxford, Oxfordshire and the General Hospital in Milton Keynes, Buckinghamshire, UK. Families were approached on the maternity wards and were invited to participate in the study, which included all family members but particularly focussed on fathers and their infants. The fathers who gave their consent received the Edinburgh Postnatal Depression Scale (EPDS)[13] at 7 weeks. Of those completing the initial screening questionnaire 12.7% scored 10 or higher. All the fathers screening positive (10 or more on the EPDS) and a random one-in-four sample of those screening negative were asked to take part in the study. Further detail on the initial screening has been published.[14] To be eligible for inclusion in the study participants were required to be age 18 or over at the time of the child's birth and to speak sufficient English to comprehend and complete the assessments. Infants' were required to have been born at no less than 37 weeks, to have a birth weight of at least 2,500 g and to have no severe illness or abnormalities.

Families were seen at home when the infant was 3 months old. All fathers (and mothers) were interviewed using the Structural Clinical Interview for DSM-IV (SCID[15]) to establish whether they met the criteria for major depressive disorder. Using the cut-off score of 10 or more, the EPDS was found to identify those with a current episode of depression with a high level of accuracy (sensitivity 89.5%; specificity 78.2%).[14] The sample for this study consisted of a group of fathers with depression (*n*=54) and a group of fathers without depression (*n*=99), based on the Structured Clinical Interview (SCID). In the depressed group, 19 had current major depressive disorder and 35 had experienced depression in the past, before the infant's birth.

### MEASURES

All measures were completed by both fathers and mothers, with the exception of assessments of antisocial traits and alcohol use which were completed by fathers only.

Major Depressive Disorder was assessed with the SCID.[15] This structured interview has been found to have satisfactory reliability and validity.

Depressive symptoms were measured with the 10 item EPDS.[13] This scale is sensitive to changes in severity of depression and has been shown to have good sensitivity (91%) and specificity (95%) in predicting depressive disorder in mothers,[16] and only marginally lower in fathers.[14,17]

Fathers' antisocial traits were measured with the Antisocial Personality Problems scale from the Adult Self-Report DSM-oriented scales. The scale consists of 20 items, with a response scale from 0=Not true to 2=Very true or often true. In this study, 5.8% of fathers scored above the cut off considered to indicate borderline or clinically significant antisocial personality problems. Internal consistency in this study was .73.

The Alcohol Use Disorders Identification Test (AUDIT)[18] measured fathers' alcohol usage. The AUDIT consists of 10 items referring to alcohol consumption, drinking behavior and problems caused by alcohol. Scores range from 0 to 40, with high scores indicating more problematic alcohol use. Previous research has shown high levels of sensitivity (88%) and specificity (81%) for dependence in men with depression or anxiety.[19]

Parents' couple relationships were measured with the Dyadic Adjustment Scale (DAS).[20] This 32-item questionnaire consists of four subscales: consensus, affectional expression, satisfaction, and cohesion. A total DAS score is obtained by summing all items of the questionnaire. Scores range from 0 to 151 with higher scores indicating a better couple relationship. High levels of internal consistency were found for both men (.89) and women (.87).

Perceived criticism in the couple relationship was assessed with two self-report items: (1) “How critical do you think you are of your partner?” and (2) “How critical do you think your partner is of you?”.[21] Responses were scored on a 10-point scale ranging from 0=Never critical to 10=Very critical indeed. Parents were also asked to rate how confident they were that their relationship would succeed, using a 0–10 scale.

Infant temperament was assessed using parents' reports on sub-scales of the Infant Behavior Questionnaire—Revised (IBQ;[22] activity level, smiling and laughter, soothability, and distress to limitations). Parents reported how often their baby showed a particular behavior in the last week on a 7-point scale ranging from 1=Never to 7=Always.

### STATISTICAL ANALYSIS

The analysis was conducted in the following stages:

1. Demographic and other clinical characteristics of depressed and nondepressed fathers were compared, including rates of depression in partners.
2. Differences in measures of couple relationship between the two groups were examined using *t*-tests. Hierarchical linear regressions were then used to investigate whether any differences found remained after adjusting for the infant's gender, father's age and education, father's antisocial traits and maternal depression, with the couple relationship as the outcome variable. The covariates infant's gender, father's age and education, father's antisocial traits and maternal depression were entered in the first step, with father's depression entered in a second step.
3. This analysis was repeated focusing only on those fathers with a current episode of depression to specifically examine whether current symptoms were more strongly associated with the dependent measures.
4. Differences in infant temperament were investigated using *t*-tests. Analyses first compared all the depressed fathers (with history and/or current depression) and then only currently depressed fathers versus the nondepressed fathers.
5. Finally, these analyses were repeated using a continuous measure of current depressive symptoms in fathers instead of a clinical diagnosis. The full range of depressive symptoms was used in this way as evidence suggests that sub-threshold symptoms of depression can be clinically important in terms of outcome.

## RESULTS

### CHARACTERISTICS OF THE SAMPLE

Fathers in the depression group had higher levels of antisocial traits and there was some evidence that they were older compared to nondepressed fathers (Table 1). Partners of the depressed fathers were older than those of the nondepressed group, and there was some evidence that they were also more likely to have a diagnosis of depression (current diagnosis and/or history of depression).

**TABLE 1:** Comparisons of parental and infant characteristics

	Depressed group (*n*=54)		Nondepressed group (*n*=99)	
		
	*M*(*SD*)	*M*(*SD*)	*t*	*P*
Father's age	36.20 (5.34)	34.47 (6.13)	−1.75	.083
Mother's age	34.40 (4.83)	32.31 (4.82)	−2.54	.012
Father's alcohol use	4.92 (3.25)	4.92 (2.58)	−0.001	.999
Father's antisocial	4.30 (3.48)	2.78 (2.54)	−2.97	.004
	%	%	χ^2^	*P*
Infant's birth order (%)			5.27	.153
First	48.1	64.6		
Second	40.7	26.3		
Third	7.4	8.1		
Fourth	3.7	1.0		
Boys (%)	59.3	45.5	2.66	.103
Maternal depression^a^	39.6	25.3	3.38	.066
Marital status (%)			2.39	.496
Married	77.4	77.3		
Living together	20.8	21.6		
Separated/Divorced	1.9	1.0		
Father's education (%)			4.71	.194
Postgraduate degree	22.6	34.7		
Degree	13.2	25.3		
Diploma	41.5	15.8		
GCSC's/A levels	22.6	24.2		
Mother's education (%)			2.31	.511
Postgraduate degree	25.5	32.3		
Degree	47.1	34.4		
Diploma	13.7	18.3		
GCSC's/A levels	13.7	15.1		

a Current and/or history of depressive disorder.

### PARENTAL COUPLE RELATIONSHIP

Fathers in the depression group reported higher levels of dissatisfaction, lower levels of affection and lower overall levels of relationship satisfaction (total scores) on the DAS compared to nondepressed fathers (Table 2). They were also less confident in the future success of their relationship, and reported higher levels of criticism (both toward and from the partner). The partners of depressed fathers reported lower levels of affection from the partner compared to the nondepressed group.

**TABLE 2:** Comparisons on measures of couple adjustment between the depressed and the non-depressed group

	Depressed group (*n*=54)	Nondepressed group (*n*=99)	*t*-Test	*P*	β*	P*
Father's report						
Affection	8.37 (2.07)	9.27 (1.84)	2.70	.008	−.176	.048
Cohesion	16.00 (3.86)	17.13 (3.46)	1.82	.071	−.126	.169
Consensus	48.29 (6.18)	49.83 (6.29)	1.35	.178	−.051	.576
Dissatisfaction	38.76 (3.15)	36.39 (4.25)	3.81	.000	−.237	.006
DAS total score	109.07 (13.11)	115.04 (11.20)	2.64	.009	−.170	.069
Mother's report						
Affection	8.69 (2.03)	9.44 (1.83)	2.26	.025	−.200	.024
Cohesion	16.17 (3.84)	16.33 (3.35)	0.26	.792	−.033	.716
Consensus	50.69 (5.62)	51.58 (5.39)	0.91	.367	−.030	.748
Dissatisfaction	38.29 (4.11)	38.66 (3.30)	0.59	.554	.033	.709
DAS total score	113.29 (11.83)	116.11 (10.03)	1.43	.156	−.088	.343
Father's report						
Success of relation	8.70 (1.26)	9.21 (0.96)	2.82	.005	−.195	.030
Critical of partner	5.28 (2.84)	4.03 (2.31)	−2.93	.004	.196	.029
Partner critical of you	5.08 (2.74)	3.88 (2.06)	−3.03	.003	.215	.017
Mother's report						
Success of relation	9.00 (1.08)	8.99 (0.95)	−0.06	.952	.079	.377
Critical of partner	4.77 (2.11)	4.76 (2.15)	−0.05	.960	−.016	.862
Partner critical of you	3.92 (2.12)	3.74 (2.19)	−0.48	.635	−.027	.775

* *B* and *P* value after adjustment for infant's gender, father's age and education, antisocial traits and maternal depression.

When hierarchical regression analyses were conducted to adjust for the potential confounding effect of infant's gender, father's age and education, antisocial traits, and maternal depression these associations remained essentially unchanged except for a weakening of the association between depression and fathers total DAS score (Table 2).

The above analysis was replicated comparing the control group (*n*=99) against only those fathers with a current episode of depression (*n*=19) (excluding fathers with a history of depression). The findings were broadly similar, with currently depressed fathers typically having lower scores on the measures of couple relationship adjustment (Table 3). In addition, currently depressed fathers were less confident in the future success of their relationship.

**TABLE 3:** Differences in couple adjustment between the currently depressed fathers and the non-depressed fathers

	Currently depressed group (*n*=19)	Nondepressed group (*n*=99)	*t*-Test	*P*	β^b^	*P*
Father's report						
Affection	7.56 (2.57)	9.27 (1.84)	3.40	.001	−.271	.007
Cohesion	15.21 (4.18)	17.13 (3.46)	2.13	.036	−.178	.090
Consensus	47.12 (5.15)	49.83 (6.29)	1.68	.097	−.078	.450
Dissatisfaction	35.47 (4.17)	36.39 (4.25)	3.78	.000	−.190	.047
DAS total score	104.00 (13.92)	115.04 (11.20)	3.39	.001	−.221	.031
Mother's report						
Affection	8.28 (2.14)	9.44 (1.83)	3.40	.018	−.259	.011
Cohesion	16.53 (3.60)	16.33 (3.35)	−0.23	.821	−.010	.924
Consensus	50.24 (5.12)	51.58 (5.39)	0.95	.345	.022	.837
Dissatisfaction	38.53 (4.05)	38.66 (3.30)	0.141	.888	.077	.464
DAS total score	113.60 (10.93)	116.11 (10.03)	0.88	.381	−.027	.809
Father's report						
Success of relation	8.52 (0.90)	9.21 (.96)	2.90	.005	−.215	.039
Critical of partner	5.26 (3.11)	4.03 (2.31)	−2.00	.047	.186	.073
Partner critical of you	5.16 (2.93)	3.88 (2.06)	−2.30	.023	.161	.117
Mother's report						
Success of relation	9.32 (0.89)	8.99 (.95)	−1.38	.170	.204	.048
Critical of partner	4.56 (2.12)	4.76 (2.15)	0.361	.719	−.058	.586
Partner critical of you	3.61 (1.72)	3.74 (2.19)	0.239	.811	−.011	.920

a DAS=Dyadic Adjustment Scale.

b Adjusted for infant's gender, father's age and education, antisocial traits and the mother's depression.

### INFANT TEMPERAMENT

There were few differences in temperament between the infants of depressed and nondepressed fathers, with only weak evidence that depressed fathers perceived their infants as more distressed compared to the nondepressed fathers (Table 4). There were no differences seen when mothers reports of infant temperament were used.

**TABLE 4:** Differences in the infant's temperament between the depressed and the nondepressed fathers

	Depressed group (*n*=54)	Nondepressed group (*n*=99)	*t*-Test	*P*
Father's report				
Distress	3.86 (0.88)	3.61 (.73)	−1.89	.061
Soothability	4.44 (0.99)	4.38 (1.06)	−0.335	.738
Smile and laughter	4.96 (0.85)	5.02 (1.04)	0.404	.687
Novelty	1.98 (1.04)	2.17 (1.29)	0.850	.397
Activity	3.39 (0.75)	3.37 (0.80)	−0.113	.910
Mother's report				
Distress	3.64 (0.88)	3.57 (0.82)	−0.443	.658
Soothability	4.69 (1.13)	4.76 (1.03)	0.398	.691
Smile and laughter	5.11 (0.99)	5.26 (1.03)	0.903	.368
Novelty	2.08 (1.46)	2.04 (1.17)	−0.160	.873
Activity	3.46 (0.74)	3.34 (0.61)	−1.11	.268

When the analyses were repeated comparing only those fathers with current depression with nondepressed fathers (Table 5), we found that those with depression reported more distress in their infants (*M*=4.25, *SD*=.88; *M*=3.61, *SD*=.73; *t*(115)=−3.38, *P*=.001). This difference was significant for both boys (*M*=4.33, *SD*=1.06; *M*=3.66, *SD*=.75; *t*(49)=−2.07, *P*=.044) and girls (*M*=4.20, *SD*=.81; *M*=3.56, *SD*=.72; *t*(64)=−2.720, *P*=.008). Using maternal reports of infant temperament, infants of currently depressed fathers had a tendency to score lower on the laughter and smiling scale (*M*=4.77, *SD*=1.08; *M*=5.26, *SD*=1.03; *t*(114)=1.89, *P*=.062). This finding was particularly marked for girls (*M*=4.42, *SD*=1.18; *M*=5.31, *SD*=.96; *t*(63)=2.79, *P*=.007).

**TABLE 5:** Differences in the infant's temperament between the currently depressed and the nondepressed fathers

	Currently depressed group (*n*=19)	Non-depressed group (*n*=99)	*t*-Test	*P*
Father's report				
Distress	4.25 (0.88)	3.61 (0.73)	−3.38	.001
Soothability	4.16 (0.98)	4.38 (1.06)	0.85	.400
Smile and laughter	4.68 (0.92)	5.02 (1.04)	1.34	.183
Novelty	2.25 (1.04)	2.17 (1.29)	−0.2	.826
Activity	3.52 (0.58)	3.37 (0.80)	−0.76	.452
Mother's report				
Distress	3.64 (1.06)	3.57 (0.82)	−0.318	.751
Soothability	4.35 (1.12)	4.76 (1.03)	1.54	.126
Smile and laughter	4.77 (1.08)	5.26 (1.03)	1.89	.062
Novelty	2.17 (1.60)	2.04 (1.17)	−.42	.679
Activity	3.58 (0.79)	3.34 (0.61)	−1.49	.139

### ANALYSES USING CONTINUOUS SCORES FOR CURRENT DEPRESSIVE SYMPTOMS IN FATHERS AND MEASURES OF FAMILY FUNCTIONING

When we repeated the analyses using continuous scores on the EPDS for depressive symptoms in fathers, they correlated with fathers' ratings of DAS affection (*r*=−.224, *P*=.002), consensus (*r*=−.200, *P*=.009), cohesion (*r*=−.204, *P*=.006), dissatisfaction (*r*=−.438, *P*=.000), and total scores (*r*=−.351, *P*<.001). When we used the mother's ratings of their relationship, high paternal depression scores correlated with the total DAS score (*r*=−.185, *P*=.019). High levels of depressive symptoms were also associated with the fathers' reports on the infant's distress temperament scale (*r*=.273, *P*<.001) and with maternal reports on the infant's activity scale (*r*=.168, *P*=.021).

## DISCUSSION

The key finding of this study is that depression in fathers in the postnatal period is associated with an increased risk of disharmony in partner relationships. This is found particularly for affection within the relationship, and importantly is reported by both men and their partners, even when controlling for maternal mood. Fathers also report higher levels of criticism in their relationship with their partner, a key predictor of more negative outcomes in relationships over time. Fathers in the depressed groups had infants with higher reported levels of distress, and this was particularly the case for fathers with current depressive disorder. Few other consistent differences in infant temperament were found.

When continuous depressive scores are used, as opposed to a clinical diagnosis, more consistent findings with parental couple disharmony, and infant temperament difficulties are found on both maternal and paternal report. Before considering the implications of these findings the strengths and limitations of the study will be considered.

### STRENGTHS AND LIMITATIONS

This study has focussed on depression affecting fathers, a generally under-researched area. Participants were recruited from routine maternity services, and structured psychiatric interviews were used, whereas almost all previous research has only used questionnaire measures of depressive symptoms. Outcome measures were reported by both mothers and fathers, allowing for a degree of independence of reporting of difficulties. Some limitations should also be noted. First, the sample of depressed fathers was modest in size, despite extensive attempts at recruitment.[14] Second, the data reported were from the same time point, and so are cross-sectional in nature. Therefore, caution should be exercised in any assumptions of direction of causality. It is possible that higher levels of partner conflict could lead to depression in either parent, rather than being the result of depression. Similarly it is possible that higher levels of infant distress could lead to increased depressive symptoms in fathers, although some studies of parental and infant symptoms point toward transmission of symptoms being predominantly in the direction from parents to children.[23] Third, the report of parental couple disharmony was by parental questionnaire, rather than an independently observed task or measure, although questionnaire report is the most commonly used approach in research, and maternal, as well as paternal, reports were used.

### IMPLICATIONS OF THE FINDINGS

This is the first study to assess the impact of a diagnosis of paternal depressive disorder on family functioning at such an early stage in an infant's life. This is of potential importance as this is a time of enormous developmental change in an infant, when it is particularly sensitive to environmental perturbations; parental psychiatric disorder and inter-parental conflict are both known to be potent examples of this.[8,24,25] Stressors or adversities experienced at this time can have significant and enduring effects on child capacities, continuing into adulthood, including an increased risk of psychiatric disorder.

Inter-parental conflict and divorce are more common in people suffering with depression, although this has primarily been explored previously in relation to women with depression.[26] Some research in families of slightly older children (attending kindergarten) has suggested that paternal depressive symptoms may be more strongly associated with couple disharmony than maternal depressive symptoms.[27] Critically, both parental depressive symptoms and inter-parental conflict independently predict an increased risk of adverse behavioral and emotional outcomes for children.[8,28] The co-occurrence of parental depression and inter-parental conflict, and the potential combined toxic effect of each on both parents and children, is of considerable concern, and yet is significantly under-researched in these first months of children's lives. There are a number of possible mechanisms by which these may affect children's development. Both parental depression and involvement in unhappy relationships are likely to lead to a degree of parental distraction and preoccupation, which will detract from each parents' ability to focus on, and meet, the needs and demands of a young baby.[29] Associated feeling of anger and dissatisfaction could also contribute to sub-optimal parenting.[30] In older children, direct exposure to parental anger and disagreement is known to be associated with increased child distress.[8] It is not clear whether this will be the case for young children, although even young infants have surprising capacities and abilities to absorb and process social information.

There were relatively few differences found between depressed and nondepressed fathers in relation to their infant's temperament, although higher levels of infant distress were reported, which may have important implications for subsequent child development. There is consistent evidence emerging of an increased risk of difficulties, particularly behavioral problems and subsequently conduct disorders, in the offspring of fathers who experience high levels of depressive symptoms.[31] This has also been found to be the case where child emotional and behavioral problems are assessed in the years following paternal depressive symptoms in the postnatal period.[5,6] However, this is the first study to assess the association between paternal depression and infant functioning so early in life, and it may be that more pronounced differences emerge later in child development; perhaps reflecting the time it may take for the impact of parental depression to emerge fully. In studies of maternal postnatal depression, it appears to be the case that chronicity and severity of maternal disorder both moderate the impact on children's development, and it is likely that this would also be the case for paternal depression.

## CONCLUSIONS

This study has demonstrated that depressive disorder affecting fathers is associated with an increased risk of inter-parental conflict in the postnatal period, and that this association still holds when maternal depression is controlled for. We also found paternal depression to be associated with somewhat higher levels of difficulties in infant temperament. This emphasizes the importance of considering seriously the potential for men, as well as women, to experience depression in the postnatal period, and the potential impact of psychiatric disorder on them, their partners and their children. The impact of parental depression, in both mothers and fathers, on the couple relationship is an area that should be at the forefront of clinicians' minds when assessing families in the postnatal period.
